# Insights into the H_2_O_2_‐driven catalytic mechanism of fungal lytic polysaccharide monooxygenases

**DOI:** 10.1111/febs.15704

**Published:** 2021-01-26

**Authors:** Tobias M. Hedison, Erik Breslmayr, Muralidharan Shanmugam, Kwankao Karnpakdee, Derren J. Heyes, Anthony P. Green, Roland Ludwig, Nigel S. Scrutton, Daniel Kracher

**Affiliations:** ^1^ Manchester Institute of Biotechnology The University of Manchester UK; ^2^ Future Biomanufacturing Research Hub Manchester Institute of Biotechnology The University of Manchester UK; ^3^ Biocatalysis and Biosensing Laboratory Department of Food Science and Technology University of Natural Resources and Life Sciences Vienna Austria; ^4^ Photon Science Institute The University of Manchester UK

**Keywords:** biomass degradation, cellobiose dehydrogenase, electron paramagnetic resonance, hydrogen peroxide, lytic polysaccharide monooxygenase, type II copper protein

## Abstract

Fungal lytic polysaccharide monooxygenases (LPMOs) depolymerise crystalline cellulose and hemicellulose, supporting the utilisation of lignocellulosic biomass as a feedstock for biorefinery and biomanufacturing processes. Recent investigations have shown that H_2_O_2_ is the most efficient cosubstrate for LPMOs. Understanding the reaction mechanism of LPMOs with H_2_O_2_ is therefore of importance for their use in biotechnological settings. Here, we have employed a variety of spectroscopic and biochemical approaches to probe the reaction of the fungal LPMO9C from *N. crassa* using H_2_O_2_ as a cosubstrate and xyloglucan as a polysaccharide substrate. We show that a single ‘priming’ electron transfer reaction from the cellobiose dehydrogenase partner protein supports up to 20 H_2_O_2_‐driven catalytic cycles of a fungal LPMO. Using rapid mixing stopped‐flow spectroscopy, alongside electron paramagnetic resonance and UV‐Vis spectroscopy, we reveal how H_2_O_2_ and xyloglucan interact with the enzyme and investigate transient species that form uncoupled pathways of *Nc*LPMO9C. Our study shows how the H_2_O_2_ cosubstrate supports fungal LPMO catalysis and leaves the enzyme in the reduced Cu^+^ state following a single enzyme turnover, thus preventing the need for external protons and electrons from reducing agents or cellobiose dehydrogenase and supporting the binding of H_2_O_2_ for further catalytic steps. We observe that the presence of the substrate xyloglucan stabilises the Cu^+^ state of LPMOs, which may prevent the formation of uncoupled side reactions.

## Graphical abstract

Lytic polysaccharide monooxygenases (LPMOs) consume external electrons and an O_2_‐containing cosubstrate to depolymerise recalcitrant polysaccharides. We show that interactions of reduced LPMO with the H_2_O_2_ cosubstrate leaves the enzyme in a reduced Cu^+^ state after a single turnover. This allows for continued catalysis without the need for external electrons. The presence of substrate stabilises the Cu^+^ state of LPMOs, which may prevent the formation of uncoupled side reactions.

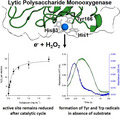

**Abbreviations**
CDH: cellobiose dehydrogenase
CYT: cytochrome domain of CDH
EPR: electron paramagnetic resonance
LPMO: lytic polysaccharide monooxygenase
*Nc*
: *Neurospora crassa*
T2Cu: type II copper
XG: xyloglucan

## Introduction

Lytic polysaccharide monooxygenases (LPMOs; EC: 1.14.99.53‐56) are copper‐dependent metalloenzymes that oxidatively cleave glycosidic bonds in recalcitrant carbohydrate polymers, disrupting their crystalline structure. LPMO substrates include chitin [1], cellulose [2, 3, 4], cellooligosaccharides [5], starch [6, 7] and various hemicelluloses [8, 9, 10]. Due to their widespread occurrence in fungi [11], bacteria [1], insects [12], plants [13] and viruses [14], as well as their ability to enhance the activity of canonical glycosyl hydrolases [2, 15], LPMOs have attracted considerable academic and industrial interest [15].

**Fig. 1:**
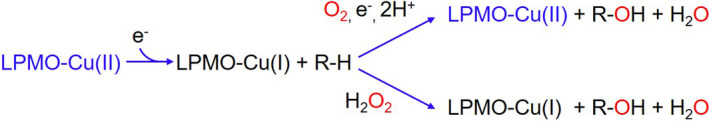
Simplified schematic of the reactions of LPMO with molecular oxygen and hydrogen peroxide. Following the reduction of the active‐site copper, LPMO interacts with an oxygen‐containing cosubstrate (oxygen or hydrogen peroxide). The interaction with O_2_ necessitates the delivery of two electrons to accomplish a full catalytic cycle. In contrast, the reaction with H_2_O_2_ requires only one external electron and leaves the active site in the reduced state following a catalytic turnover.

All known LPMOs, irrespective of origin, share highly similar active‐site geometries, in which two histidine residues coordinate a single type II copper ion (T2Cu) in a conformation commonly referred to as the ‘histidine brace’ [4]. As of yet, the detailed catalytic mechanism of LPMOs remains uncertain. In particular, the identity of the reactive oxygen intermediate that facilitates polysaccharide cleavage is still a topic of discussion and debate [16]. LPMO activation is achieved by reduction of the T2Cu by a redox partner to generate the Cu^+^ form of the enzyme (Fig. 1). Once reduced, an oxygen‐containing cosubstrate (molecular oxygen [17, 18] or H_2_O_2_ [19, 20]) binds to and is activated by the enzyme (Fig. 1). Following these steps, it is thought that either a superoxo [21] or oxyl species [22, 23] forms at the T2Cu site to facilitate the regioselective hydroxylation of either the C1 or C4 carbon present in the polysaccharide substrate, thereby destabilising and breaking the glycosidic bond [16]. Several studies have demonstrated the activation of molecular oxygen [4, 17, 18, 24] at the LPMO active site. It has also recently been shown that H_2_O_2_ is an efficient cosubstrate for LPMOs [19, 20, 25], although its relevance in the native environment is currently an area of debate [26, 27]. As the catalytic efficiencies that can be achieved in H_2_O_2_‐driven LPMO reactions are several orders of magnitude higher compared to the reaction with molecular oxygen [28], the role of H_2_O_2_ in the catalytic mechanism of LPMO is of high interest and is of importance for its use in industrial settings [15, 29]. A commonly discussed drawback of H_2_O_2_‐mediated LPMO reactions is the instability of LPMOs under suboptimal reaction conditions, for example upon overfeeding with H_2_O_2_ or under substrate depletion [19, 30]. To date, a range of computational and experimental studies [19, 20, 25, 31, 32, 33, 34] support a general peroxygenase reaction of LPMOs and have provided a synoptic view on the H_2_O_2_‐dependent reaction mechanism.

While the reaction pathways of the O_2_‐ and H_2_O_2_‐mediated LPMO catalysis are hypothesised to proceed through a common intermediate [34], a notable difference between the two pathways is the consumption of electrons and protons during the reaction. For the LPMO reaction with molecular oxygen, two electrons and two protons are required from a redox partner to form the putative reactive copper‐oxo species (Fig. 1). In this scenario, the initial reduction of the T2Cu and activation of dioxygen would need to be followed by a well‐timed delivery of the two protons and the second electron [21]. In contrast, the interaction of reduced LPMO with the 2‐electron reducing H_2_O_2_ would directly lead to the formation of a radical intermediate and supply the necessary electrons and protons required for the reaction (Fig. 1) [19, 25]. When H_2_O_2_ is used as a cosubstrate, it is thought that the T2Cu centre would remain in the Cu^+^ state following catalysis. In this reduced form, the LPMO is competent to undergo subsequent turnover reactions without the need for an electron from a redox partner. Measurements of the bacterial LPMO CBP21 have indeed shown that the enzyme could perform multiple (up to 18) turnovers with H_2_O_2_ after a single ‘priming’ reduction in the catalytic site [31], although direct spectroscopic evidence of the reduced form following LPMO turnover when H_2_O_2_ is used as a substrate is currently lacking.

The well‐studied fungal LPMOs obtain electrons from lignin‐derived phenolic reductants [35, 36, 37, 38] or specific partner redox enzymes, such as the flavocytochrome cellobiose dehydrogenase (CDH) [21, 39] or the PQQ‐dependent pyranose dehydrogenases [40]. Importantly, both of these enzymes feature a flexible electron‐transferring cytochrome domain, which activates the LPMO through the transfer of single electrons to the T2Cu [39, 41]. NMR and computational studies [42, 43] have shown that cellulosic substrates and the cytochrome domain of CDH interact with the same patch on the LPMO surface surrounding the T2Cu. In the substrate‐bound state of LPMO, it is therefore questionable whether the cytochrome domain would be able to access the T2Cu to achieve the delivery of the required second electron when oxygen is used as a cosubstrate. In an H_2_O_2_‐driven reaction, it is feasible that the initial reduction of the T2Cu by CDH occurs in the substrate‐free form of the protein, while cosubstrate binding and consecutive catalytic cycles could proceed in the substrate‐bound state, or when LPMO is in proximity to the substrate. This is consistent with the observation that the reduction of the T2Cu in LPMOs enhances their affinity for substrates, as was shown for both fungal [33, 44, 45] and bacterial LPMOs [31, 32]. Substrate binding also exerts a stabilising effect on LPMOs [19, 30, 44] and may enable the enzyme to tightly control cosubstrate activation, which is important to maintain catalytic stability [46, 47]. In particular, it was shown that under conditions of substrate depletion or in the presence of excess H_2_O_2_, LPMO undergoes a range of uncoupled reactions leading to oxidation of amino acids in the vicinity of the T2Cu or even to proteolytic degradation [19, 30, 48]. Most fungal LPMOs contain a tyrosine in the vicinity (~3.5 Å) of the copper, which is frequently substituted by phenylalanine in bacterial LPMOs [49]. The recently reported formation of amino acid radicals in minor reaction pathways [20, 48, 50] is strong evidence for a ‘hole‐hopping’ pathway through aromatic amino acids [51] in LPMO, which protects the active site from such autooxidation reactions.

As the majority of LPMO substrates are insoluble, it has been challenging to study the reaction mechanism of these enzymes and thus understand how they catalyse the depolymerisation of polysaccharides. In this study, we have taken advantage of the ability of the fungal LPMO9C from *Neurospora crass*a (*Nc*LPMO9C) to cleave the soluble polysaccharide xyloglucan (XG) [8], allowing us to investigate several key details surrounding the catalytic mechanism of fungal LPMOs. XG is a heterogenous hemicellulosic polymer of ca. 225 kDa, which consists of a β‐1,4‐linked glycan backbone substituted with 1,6‐linked xylose residues. Previous investigations have shown that *Nc*LPMO9C efficiently binds (*K*_d_ of 2.3 ± 0.5 µm [52]) and degrades unbranched glucosyl residues within XG through oxidation at the C4 position [8, 53]. During the degradation reaction, LPMO requires a source of reduction equivalents (from a partner protein or small molecule reductants) and a steady supply of the cosubstrate (dioxygen or H_2_O_2_) to generate oligomers with lower molecular masses [53]. Herein, to further understand the reaction mechanism of fungal LPMOs, we investigate how the cosubstrate H_2_O_2_ and the XG substrate interact with the fungal *N. crassa* LPMO9C and support catalysis.

## Results and Discussion

### A single electron priming reaction activates catalysis in fungal LPMOs when H_2_O_2_ is used as a cosubstrate

There has been much debate and discussion about the role of H_2_O_2_ in the reaction mechanism of LPMOs. Activation of the fungal enzymes is thought to be attained by reduction of the type II copper (T2Cu) site by a redox partner protein (e.g. cellobiose dehydrogenase; CDH) and a steady supply of a cosubstrate [33]. However, little is known about the stoichiometry of this reaction and how H_2_O_2_ and redox partner proteins interact with LPMO to drive polysaccharide depolymerisation. Therefore, in this study, we first set out to quantify both the reduction equivalents and the cosubstrate molecules consumed by the fungal *Nc*LPMO9C during the H_2_O_2_‐dependent degradation of XG to gain insights into the steady‐state mechanism of fungal LPMOs.

**Fig. 2:**
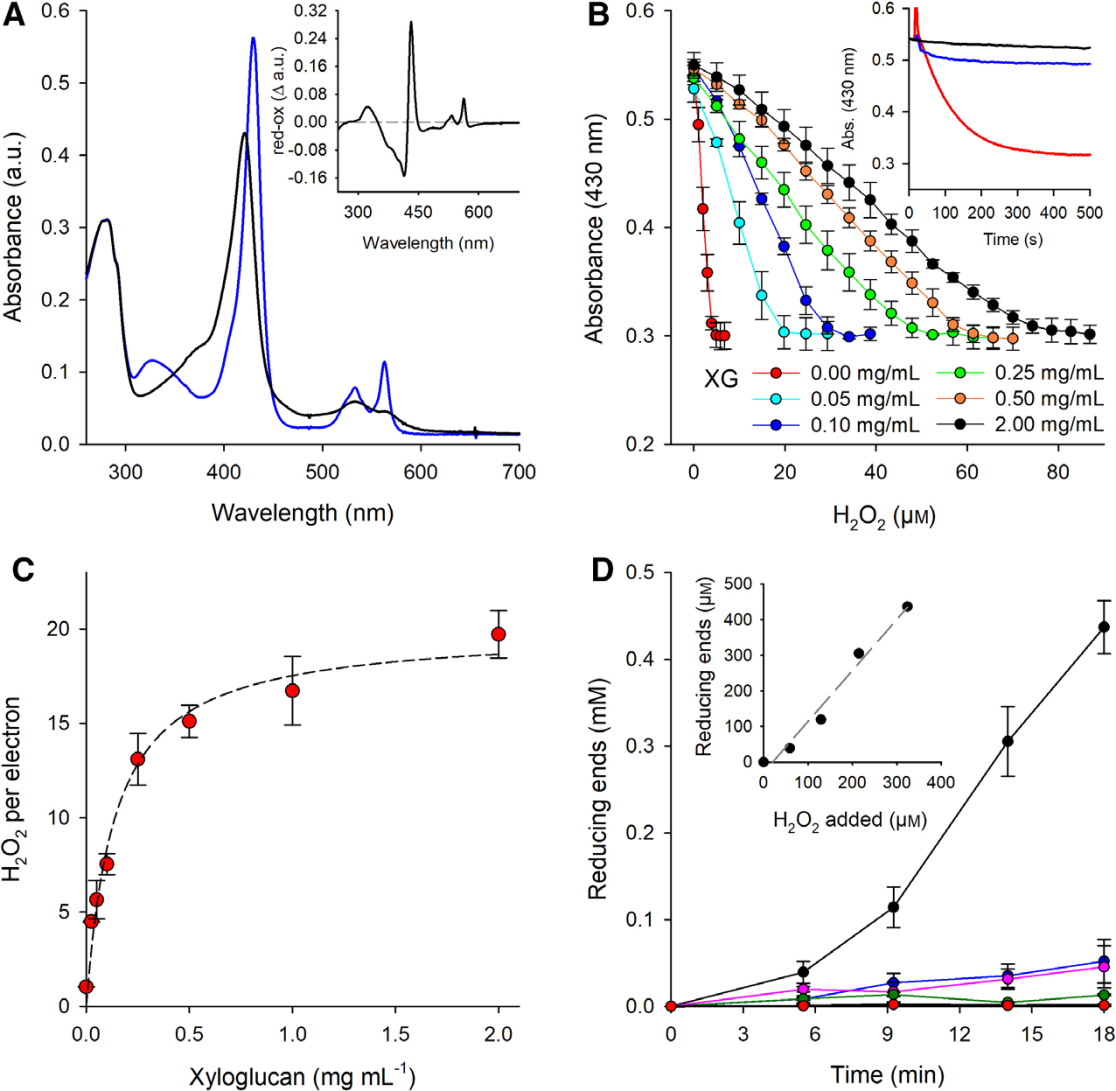
A single electron priming reaction supports up to 20 catalytic cycles of *Nc*LPMO9C with the peroxide cosubstrate. (A) UV‐Vis spectra of the cytochrome domain from *N. crassa* CDHIIA (*Nc*CYT) in its oxidised (black) and reduced state (blue). The inset shows the differential spectrum (reduced–oxidised). The experimentally determined molar absorption coefficient at 430 nm of 124 mm ^−1^ cm^−1^ in the reduced form of the enzyme was used to calculate the electrons transferred from the *Nc*CYT to the LPMO. (B) Reoxidation of *Nc*CYT (3.5 µm) by H_2_O_2_ in the presence of LPMO (0.5 µm) and varying concentrations of xyloglucan measured at 430 nm. The inset shows the time‐dependent reoxidation of 3.5 µm reduced CYT in buffer (black line) and upon addition of 10 µm of H_2_O_2_ in the presence (blue line) or absence (red line) of 0.5 µm LPMO. Samples contained 2 mg mL^−1^ of XG. (C) The required H_2_O_2_ concentration to fully reoxidise *Nc*LPMO9C as a function of the xyloglucan concentration. All experiments were performed in an anaerobic glove box at a constant temperature of 25 °C. Error bars show the mean of three replicates ± SD. (D) Determination of reducing ends during the degradation of xyloglucan by LPMO. Assays contained 1 µm LPMO, 2 mg mL^−1^ XG and 0.2 mm ascorbate. H_2_O_2_ was added in 20 µm aliquots approximately every 60 s (black circles). Control reactions carried out under the same condition by adding buffer instead of H_2_O_2_ (blue circles) were subtracted from these data. In additional control experiments, LPMO (pink circles), ascorbate (green circles) or XG (red circles) was omitted. Samples were taken regularly, and reducing ends were determined with the Somogyi–Nelson assay using the xyloglucan heptasaccharide X_4_C_3_ as calibration standard (see materials and methods). Error bars show the mean of three replicates ± SD.

Under physiological conditions, it is understood that the reduced haem *b* present in the CDH flavocytochrome supplies electrons to the T2Cu centre in *Nc*LPMO9C. For simplicity, we utilised the isolated cytochrome domain (CYT) of *N. crassa* CDHIIA to supply electrons to *Nc*LPMO9C in this investigation [21, 39, 54]. Previously published data have shown that CYT is responsible for electron transfer to the LPMO protein [39]. In Fig. 2A, the absorbance spectra of the fully reduced and oxidised *N*. crassa CDH cytochrome domain (*Nc*CYT) are shown. As with many haem proteins, there is a noticeable change in the absorbance spectral properties of the *Nc*CYT domain when transitioning from an oxidised (ferric) state to a reduced (ferrous) state (Fig. 2A). We used these changes in the haem *b* absorbance in our investigation to monitor and accurately quantify the transfer of electrons from *Nc*CYT to the LPMO protein in the presence of varying concentrations of H_2_O_2_ and XG. With a goal of understanding the roles of H_2_O_2_ and the CDH partner protein during catalysis, we first mixed reduced *Nc*LPMO9C with an excess of reduced *Nc*CYT under anaerobic conditions. Initially, H_2_O_2_ was added to the reduced species in the absence of substrate. Under these conditions, stoichiometric amounts of H_2_O_2_ were required to completely reoxidise the system. Expectedly, 4.1 µm of H_2_O_2_ had to be added to the reaction to reoxidise 3.5 µm of *Nc*CYT and 0.5 µm of *Nc*LPMO9C, which corresponds to a ratio of electrons:H_2_O_2_ of approximately 1:1, therefore demonstrating that H_2_O_2_ is an efficient oxidant for *Nc*LPMO9C in the absence of suitable substrates. Next, we tested the effect of XG on the supply of electrons from *Nc*CYT and the requirement of H_2_O_2_ during the steady‐state turnover of *Nc*LPMO9C. In Fig. 2B, the H_2_O_2_ titrations with different amounts of added XG are presented. These data show that the amount of H_2_O_2_ required to achieve full reoxidation of *Nc*CYTIIA and *Nc*LPMO9C increased as more of the XG substrate was added to the reaction mix. At the highest employed XG concentration of 2 mg·mL^−1^, approximately 80 µm of H_2_O_2_ had to be added to the reaction to fully reoxidise 3.5 µm of *Nc*CYTIIA and 0.5 µm of *Nc*LPMO9C (approx. 4 µm of electrons). It has to be noted that H_2_O_2_ slowly reoxidised the CYT domain in the absence of LPMO (Fig. 2B, inset). However, in the presence of LPMO, only a minor fraction of CYT was reoxidised upon addition of H_2_O_2_. This absorbance change occurred within the mixing time of the reaction and indicates that the H_2_O_2_ added was consumed by the LPMO and not the CYT domain under the reaction conditions used. From the slopes of the titration plots presented in Fig. 2B, the ratio of H_2_O_2_ consumed for each electron supplied from *Nc*CYT could be calculated. It was shown that at saturating XG concentrations, 20 ± 1 molecules of H_2_O_2_ were consumed per electron ‘priming’ reaction (Fig. 2C).

To further our analysis and verify that the added H_2_O_2_ was used for the catalytic cleavage of xyloglucan, we quantified the reducing ends introduced into the substrate by the LPMO reaction. LPMO catalysis (oxidation of the C4 carbon in the glycosidic bond) generates a new reducing end after each catalytic turnover. These reducing ends can be quantified using the Somogyi–Nelson assay, which can be used to monitor LPMO turnover. In these experiments, 0.2 mm ascorbate was used as a reducing surrogate that can supply electrons to *Nc*LPMO9C, and H_2_O_2_ was manually added to the reaction at a rate of approximately 20 µm·min^−1^. We must note that electron ‘priming’, which was measured in this assay by monitoring ascorbate consumption with an experimentally determined extinction coefficient of 2.82 mm ^−1^ ·cm^−1^ at 290 nm, was within the same range as that recorded when *Nc*CYT was used as a surrogate electron donor (Fig. 2B). Through the use of the Somogyi–Nelson assay, we determined that the formation of reducing ends from XG correlated with the addition of H_2_O_2_ (Fig. 2D), but the amount of measured reducing ends was slightly higher than the H_2_O_2_ added. In total, 330 µm of added H_2_O_2_ formed approximately 437 µm of reducing ends. Since the detailed product profile of the *Nc*LPMO9C reaction is not known, the lack of a suitable calibration standard may explain the discrepancy between the added H_2_O_2_ and the measured concentration of reducing ends (e.g. we calibrated the reducing end assay with the XG‐derived heptasaccharide X_4_C_3_, which gave a 30% higher signal than glucose at the same concentration). Control reactions containing ascorbate and *Nc*LPMO9C in the absence of H_2_O_2_ showed a comparatively low enzyme turnover (52 µm reducing ends were detected at the end of the assay) demonstrating both H_2_O_2_ and the ‘priming’ electron are required for catalysis.

Taken together, these results show that H_2_O_2_ can act as a cosubstrate for the cleavage of XG by *Nc*LPMO9C (up to now not shown for other LPMOs) and that only substoichiometric amounts of electrons are required for the ‘priming’ of LPMOs for subsequent H_2_O_2_‐driven catalysis. Analogous to the bacterial, chitin‐degrading LPMO10 from *Serratia marcescens*, which was shown to catalyse up to 18 turnovers per electron [19, 32], we here observe that a singly reduced fungal LPMO can catalyse multiple turnovers without requiring additional reduction equivalents. With this understanding of the role of the redox partner protein and H_2_O_2_ in the steady‐state reaction of fungal LPMOs, we set out to understand and identify catalytic intermediates in the reaction cycle of *Nc*LPMO9C using H_2_O_2_ as a cosubstrate and XG as a substrate (*vide infra*).

### NcLPMO9C catalysis with H_2_O_2_ causes the T2Cu centre to remain in a Cu(I) state following xyloglucan degradation

**Fig. 3:**
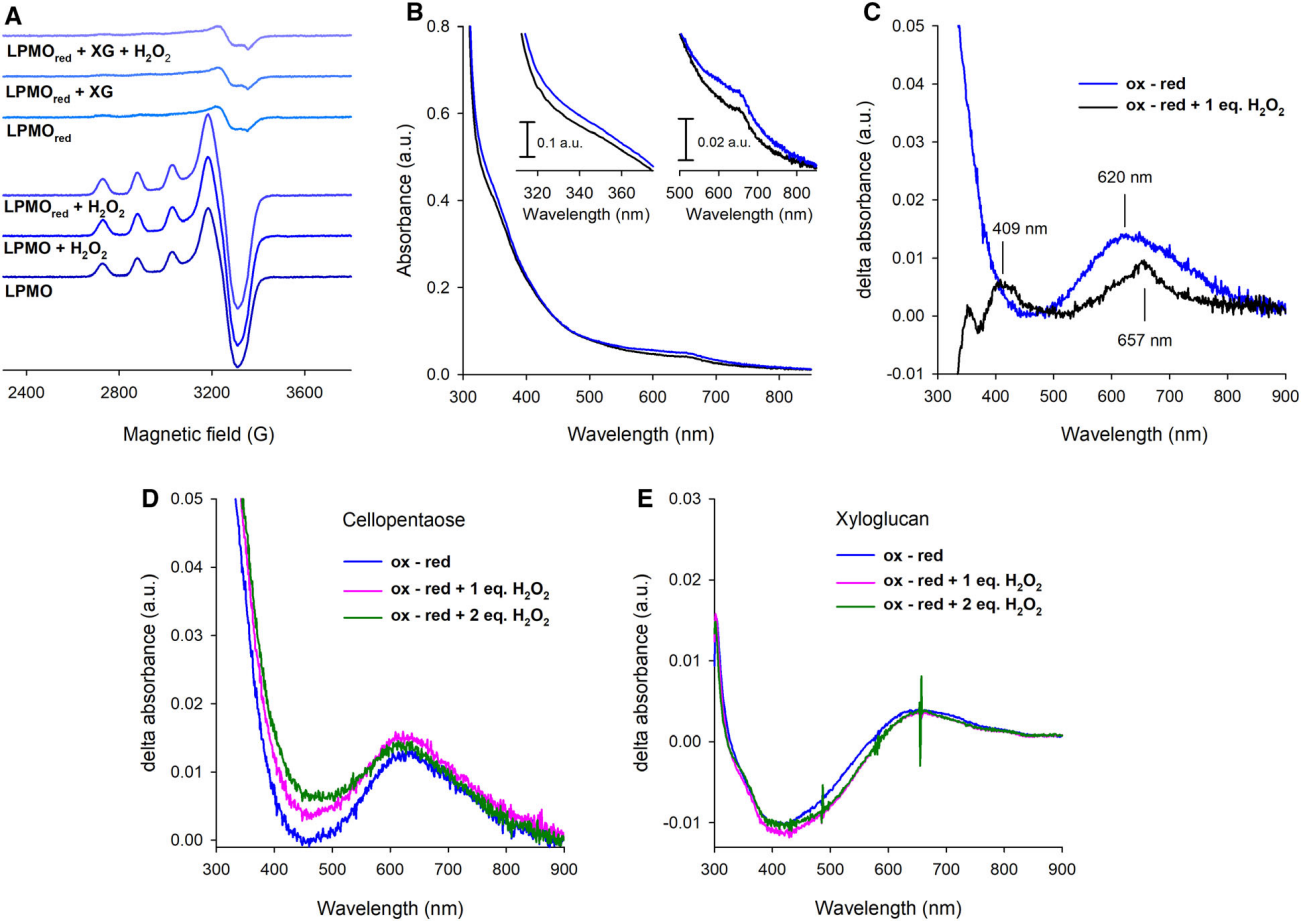
Redox states of the T2Cu centre present in *Nc*LPMO9C when peroxide is used as a cosubstrate. (A) EPR spectra of oxidised and reduced LPMO (300 µm) in the presence and absence of xyloglucan (XG, 3.3 mg mL^−1^). All samples were prepared in an anaerobic glove box and immediately frozen in liquid N_2_. (B) UV‐Vis spectra of *Nc*LPMO9C (300 µm) in the oxidised (blue line) and ascorbate‐reduced state (black line) (C) Differential (oxidised‐reduced) UV‐Vis spectrum of 300 µm LPMO (blue line) in the absence of substrate. Addition of H_2_O_2_ (300 or 600 µm) to this reaction did not cause reformation of the Cu^2+^ state in LPMO. Differential (oxidised‐reduced) UV‐Vis spectrum of LPMO in the presence of (D) 5 mm cellopentaose or (E) 5 mg mL^−1^ XG. Note that the latter experiment, (E), was carried out in the presence of 150 µm LPMO due to the high viscosity of the solution. All spectra were recorded in an anaerobic glove box at a temperature of 30°C.

To further understand how only a single ‘priming’ electron transfer reaction from CDH is sufficient to catalyse multiple turnovers with the H_2_O_2_ cosubstrate, we monitored the redox state of *Nc*LPMO9C at different stages of catalysis using electron paramagnetic resonance (EPR) and UV‐Vis spectroscopic methods. In the Cu^2+^ state, the T2Cu site present in LPMOs can be visualised by EPR spectroscopy (Fig. 3A). EPR can be used to monitor the redox state of the T2Cu, providing insights into the catalytic mechanism of LPMOs. To investigate the redox state of the T2Cu present in *Nc*LPMO9C during turnover, we first used ascorbate to fully reduce the T2Cu site in the absence of substrate under anaerobic conditions. As expected, a disappearance of the EPR signal, attributed to the Cu^+^ state of the T2Cu centre, was observed when adding stoichiometric amounts of electrons to oxidised *Nc*LPMO9C. When one‐electron reduced LPMO was reacted with approx. 1.2 equivalents of H_2_O_2_, we observed that the signal attributed to the Cu^2+^ reformed (Fig. 3A), suggesting a full reoxidation of the T2Cu site following the reaction with H_2_O_2_. In Fig. 3B, the absorption spectra of oxidised and 1‐electron, ascorbate‐reduced *Nc*LPMO9C are shown. As previously demonstrated [55], there is a decrease in the 620 nm T2Cu absorbance feature when LPMO is stoichiometrically reduced from a Cu^2+^ state to a Cu^+^ state (Fig. 3C). While EPR shows the complete reformation of the Cu^2+^ signal, our UV‐Vis data for the reaction between reduced *Nc*LPMO9C and 1.2 equivalents of H_2_O_2_ show the formation of new species with spectral features at 657 and 409 nm. Recently, a number of reports have shown that LPMO can react with H_2_O_2_ in the absence of substrate [20, 48, 50], generating several amino acid radicals, which are thought to form part of a protective ‘hole‐hopping’ pathway [51]. While the reduced T2Cu site does appear to fully oxidise under these conditions (Fig. 3A), our data indicate that the reaction of reduced *Nc*LPMO9C with H_2_O_2_ in the absence of substrate generates an alternative form of the oxidised T2Cu site. While further investigation is required to characterise this species, we hypothesise that these spectroscopic features are generated by radical species in ‘hole‐hopping’ pathways, which could be associated with changes in the local environment surrounding the T2Cu site.

Next, we induced a full catalytic cycle by addition of H_2_O_2_ to reduced *Nc*LPMO9C in the presence of XG (3.3 mg·mL^−1^). First, *Nc*LPMO9C was stoichiometrically reduced with ascorbate in the presence of XG. A catalytic reaction of the enzyme was initiated by adding 1.2 equivalents of H_2_O_2_, ensuring that the LPMO could perform only a single catalytic turnover. Analysis of this reaction by EPR showed that the active‐site copper remained in the Cu^+^ state following a single turnover (Fig. 3A). The same effect could be observed by UV‐Vis spectroscopy. Specifically, the 620 nm signal attributed to the Cu^2+^ state of the T2Cu site did not reform in the enzyme when reacted with equimolar amounts of H_2_O_2_ and ascorbate in the presence of the substrate cellopentaose (Fig. 3D) or xyloglucan (Fig. 3E). Overall, these experiments provide direct spectroscopic evidence that *Nc*LPMO9C leaves a catalytic cycle in the reduced form (Cu^+^). The formation of this redox state following turnover is beneficial for catalysis as, in this state, the enzyme is capable of binding another H_2_O_2_ molecule, it has a higher affinity for the substrate [44, 45] and does not require further reduction by the redox partner cellobiose dehydrogenase.

### Substrate binding modifies the redox properties of the NcLPMO T2Cu centre but does not stimulate interprotein electron transfer from CDH

**Fig. 4:**
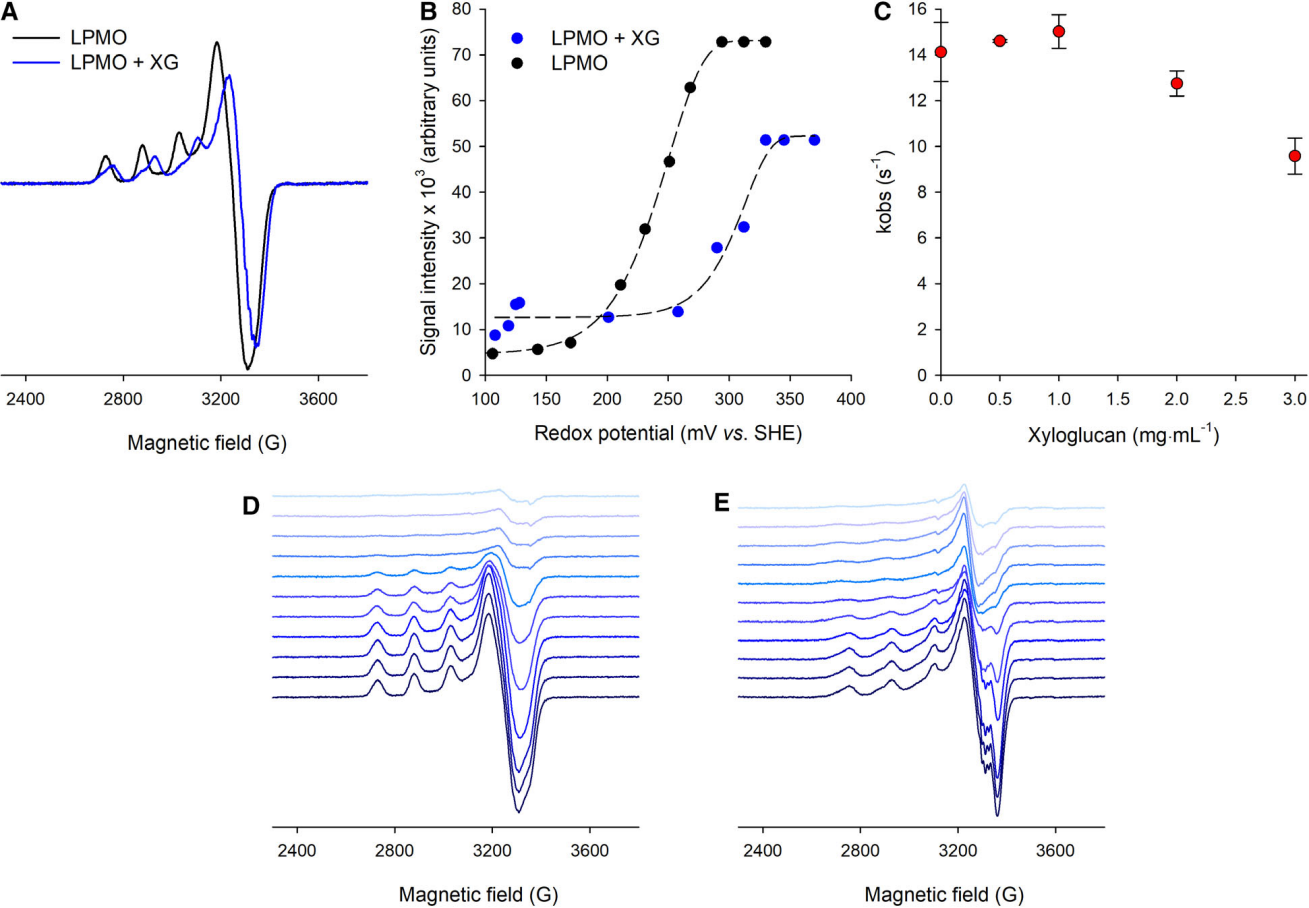
Substrate binding modifies the redox potentials of the T2Cu site in *Nc*LPMO. (A) EPR spectra (20 K) of *Nc*LPMO9C (150 µm) in the absence (black trace) and presence (blue trace) of xyloglucan (3.3 mg mL^−1^). (B) EPR redox titrations of *Nc*LPMO9C with ascorbate in the absence (black circles) and presence of 3.3 mg mL^−1^ XG (blue circles). Data in (B) are fit to the Nernst equation. (C) Effect of xyloglucan on the rate of electron transfer between CDH and LPMO. In (C), CDH (2 µm) was stoichiometrically reduced by cellobiose and mixed with an excess of *Nc*LPMO9C (12.5 µm) in a stopped‐flow device. Reoxidation of the haem *b* was measured at 430 nm. Experiments were carried out in an anaerobic glove box at a temperature of 30°C. EPR spectra of the redox titration in the (D) absence and (E) presence of the XG substrate. Experimental conditions are the same as in Fig. 3A.

Experimental [42, 44] and computational studies [43] have indicated that the interaction of LPMOs with their substrates involves protein conformational changes, which may alter the local environment around the copper centre. Specifically, the loop regions in the vicinity of the active site are thought to change conformation when the substrate binds to the active site. Changes in the active site surrounding the copper ion are also likely to change the redox potential of the T2Cu. Likewise, several studies have shown that active‐site reduction may induce such changes, which could explain why LPMOs show a higher affinity to their substrates in the reduced state [44, 45]. Here, to further understand how XG binding influences the chemistry of the T2Cu present in *Nc*LPMO9C, we measured the redox potential of the T2Cu in the absence and presence of XG using EPR spectroscopy. As shown in Fig. 4A, and as previously reported, when XG binds to the catalytic pocket of LPMO, the T2Cu EPR signal is altered (*g*_para_ shifts from 2.267 in the absence of substrate to 2.226 in the presence of XG) [52]. We used the intensity of the Cu^2+^ signal in the presence and absence of XG to report on the midpoint potential of the T2Cu site when titrated with ascorbate under anaerobic conditions (Fig. 4B). Our data show that in the absence of substrate, the redox potential for the [Cu^2+^]/[Cu^+^] couple in the *Nc*LPMO9C is 241 ± 2 mV (vs. SHE) (Fig. 4B), a result that is in excellent agreement with those previously published using UV‐Vis spectroscopic methods [52]. In the presence of XG, there is a significant increase in the midpoint potential for the T2Cu site of approximately 64 mV to 305 ± 6 mV (vs. SHE) (Fig. 4B).

An increase in the redox potential upon substrate binding is common in redox enzymes, and in some cases, it can stimulate intra/interprotein electron transfer events (e.g. in P450 proteins [56]). Moreover, modifications in redox potential upon substrate binding are also seen in a variety of redox proteins, such as DNA photolyase [57] and UDP‐galactopyranose mutase [58], and may help stabilise the reduced forms of *Nc*LPMO9C from oxidants, thus reducing uncoupled side reactions. Here, we determined whether the redox changes observed in *Nc*LPMO9C in the presence of XG stimulated electron delivery from the enzyme redox partner, CDH, by rapid mixing stopped‐flow spectroscopy. Figure 4C shows the observed rates of electron transfer from the reduced CDH protein to *Nc*LPMO9C in the presence of XG. Across the substrate concentrations tested (0–3 mg·mL^−1^), there was no significant change in the rate of electron transfer from the reduced haem *b* of CDH to the copper centre present in *Nc*LPMO9C. As the substrate‐binding site and the site of the LPMO‐partner protein interaction are thought to be the same [43, 59], it is often assumed that substrate binding prevents interactions with redox partner proteins due to steric hindrance. XG is a relatively large LPMO substrate, and we do indeed observe a slight reduction in the observed rate of electron transfer from CDH to LPMO as the substrate concentration is increased (Fig. 4C), suggesting that substrate binding slightly reduces the efficiency of interprotein electron transfer. Based on these data, we conclude that the redox potential shift upon substrate binding at the T2Cu site in *Nc*LPMO9C does not serve to enhance interprotein electron delivery from partner proteins but may be involved in the stabilisation of the Cu^+^ state for catalytic turnover, reducing any potential uncoupled side reactions occurring.

### Transient species produced by NcLPMO9C when H_2_O_2_ is used as a cosubstrate

**Fig. 5:**
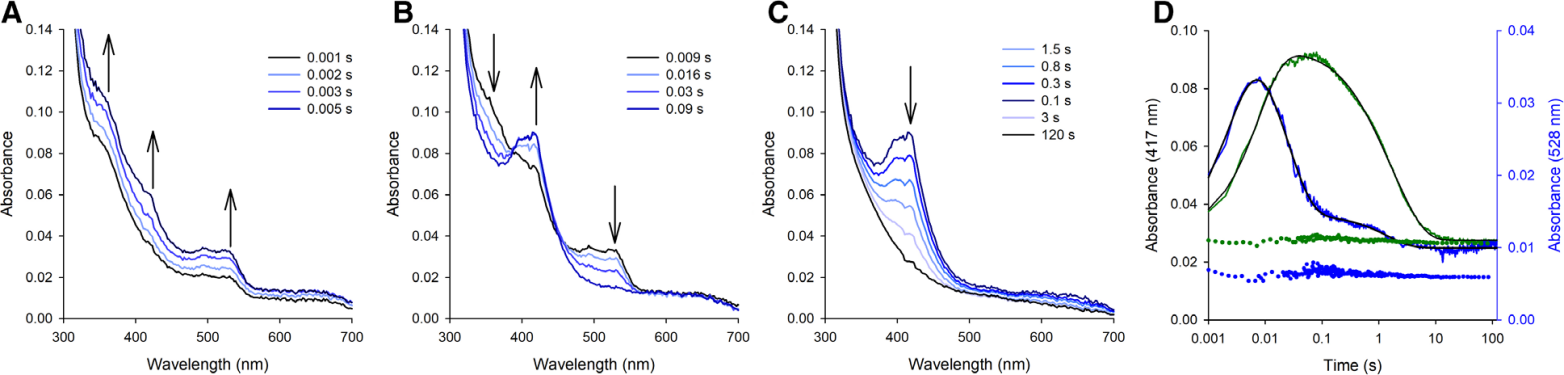
Transient species formed in *Nc*LPMO when peroxide is used as a cosubstrate. The reaction of reduced *Nc*LPMO9C (50 µm final concentration) with a 50‐fold excess of H_2_O_2_. (A) The formation of the 528 nm feature was observed within 5 ms. (B) The slower decay of 528 nm feature and the formation of the 417 nm intermediate. (C) The decay of the 417 nm feature occurred within several seconds and led to the reoxidation of the T2Cu in *Nc*LPMO9C and formation of the species seen in Fig. 3C. The enzyme was stoichiometrically reduced with 25 µm ascorbate at pH 6 (100 mm sodium phosphate buffer) in an anaerobic glove box and mixed with a 50‐fold excess of H_2_O_2_. Reactions were carried out at 4°C. (D) Stopped‐flow transients of the 528 nm feature (blue line) and the 417 nm feature (black line) in the absence of XG. Black lines show the fit used to derive the kinetic constants. Traces recorded in the presence of 2 mg mL^−1^ are shown as green (417 nm) or blue (528 nm) dots.

In a recently published report, it was shown that a number of amino acid radicals are generated in the fungal *Hypocrea jecorina* LPMO9A (*Hj*LPMO9A) when the reduced form of the enzyme was reacted with H_2_O_2_ in the absence of substrate [20]. The formation of these amino acid radicals was accredited to two separate minor uncoupled reaction pathways that occur in the absence of substrate [20] and are likely to form part of a ‘hole‐hopping’ pathway [20, 48, 51] that diverts holes from the LPMO active site to prevent oxidative damage to the T2Cu. In *Hj*LPMO9A, a Trp residue (defined by a 520 nm spectral feature) and a Tyr residue (defined by a 420 nm spectral feature) that are in close proximity to the T2Cu are hypothesised to be the source of these radical species. Like many other fungal LPMOs, this proximal Trp residue is substituted with a redox inactive Ile in *Nc*LPMO9C [60]. To understand the differences in the transient state kinetics and the radical intermediate species that form in these two forms of fungal LPMOs, we reacted 1‐electron reduced *Nc*LPMO9C with a 50‐fold excess of H_2_O_2_ under anaerobic conditions in a stopped‐flow device. In Fig. 5, stopped‐flow data for this reaction are presented. We observed that within 100 ms, two distinct spectroscopic features with maxima at 528 nm and 417 nm were formed: the spectral feature at 528 nm formed within 0.01 sec (*k*_1_ = 372 ± 26 s^−1^) and rapidly decayed (*k*_2_ = 37 ± 3 s^−1^), whereas the 417 nm feature formed slower (*k*_1_ = 114 ± 2 s^−1^) and remained stable for several seconds, decaying in a biphasic manner (*k*_2_ = 2.92 ± 0.38 s^−1^ and *k*_3_ = 0.27 ± 0.01 s^−1^). The presence of substrate (XG) suppressed the formation of both intermediates in *Nc*LPMO9C (Fig. 5D), as was also reported for *Hj*LPMO9A [20], suggesting the transient species may be either noncatalytic or may decay faster than they form in the presence of substrate due to changes in redox potential (*vide supra*) or conformational changes around the T2Cu site.

**Fig. 6:**
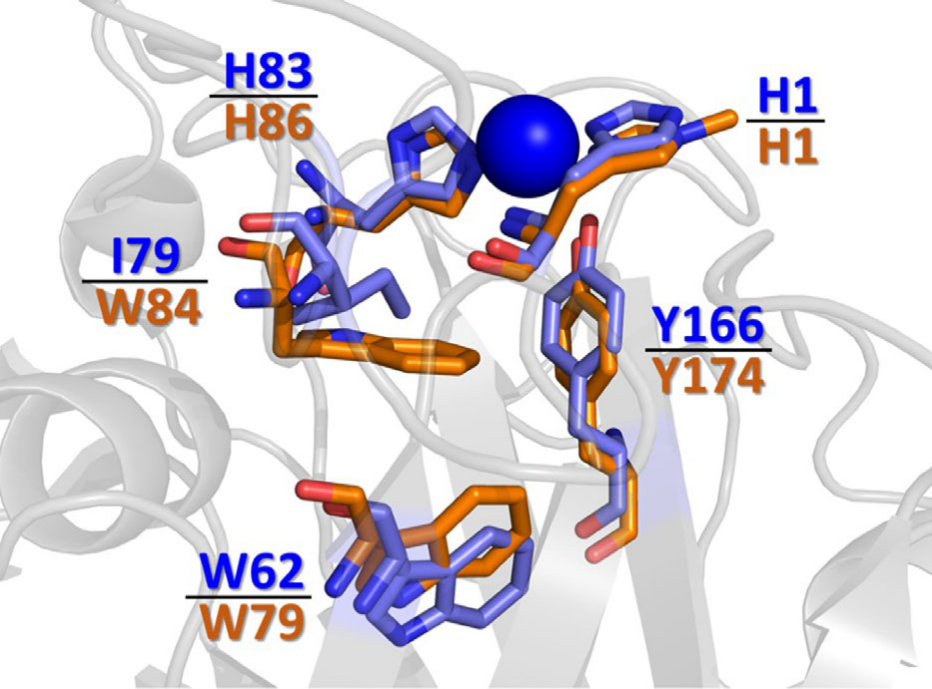
Structural overlay of the crystal structures of *Nc*LPMO9C (blue, PDB‐ID: 4D7U) and *Hypocrea jecorina* LPMO (orange, PDB‐ID: 5O2W). Note that H1 in *Hj*LPMO is methylated. The figure was generated with PyMOL v2.4 (Schrödinger, Inc.).

These observed spectroscopic species for the reaction between reduced *Nc*LPMO9C and H_2_O_2_ are in excellent agreement with a previously reported study on *Hj*LPMO9A [20], suggesting identical intermediates are formed in these two enzymes. Assuming molar absorption coefficients of 1.9 mm ^−1^ ·cm^−1^ and 2.6 mm ^−1^ ·cm^−1^ for the tryptophanyl and tyrosyl radical [20], respectively, we estimate that the tryptophanyl radical was formed in 20% of the *Nc*LPMO9C enzymes, while the tyrosyl radical was formed in 50% of the enzymes when reacted with H_2_O_2_. Of note, these proportions are higher than those observed in *Hj*LPMO9A (~30% of the total reaction) [20]. In Fig. 6, a comparison of the *Hj*LPMO9A and *Nc*LPMO9C active‐site structures is presented. There is a high level of similarity between these two enzymes. The copper‐coordinating histidine residues (His1 and His83 in *Nc*LPMO9C) and the axial tyrosine (Tyr166 in *Nc*LPMO9C) are highly conserved in both active sites (Fig. 6). The only significant difference between the active sites of *Nc*LPMO9C and *Hj*LPMO9A is the substitution of the proximal Trp residue by an Ile in *Nc*LPMO9C. This Trp is suggested to form the radical species in *Hj*LPMO9A. As the spectral features (and intensity) of this 528 nm intermediate are highly similar to those previously reported for Trp radicals, we searched for Trp residues that are in close proximity to the T2Cu site in both *Hj*LPMO9A and *Nc*LPMO9C. The tryptophan residue closest to the active‐site copper of *Nc*LPMO9C is Trp62, which is 10.7 Å away from the T2Cu, making it a candidate for the radical Trp. Based on these data, we surmise that either Trp62 forms this radical species in *Nc*LPMO9C (and possibly *Hj*LPMO9A) or that the origin of the 528 nm species can be accredited to an alternative source. However, we note that further experimental evidence is required to assign the 528 nm species in *Nc*LPMO9C.

## Conclusions

The role of hydrogen peroxide in the native environment of fungal LPMOs is currently an area of debate and discussion. However, numerous reports now detail that H_2_O_2_ can be used as an efficient oxidant for the depolymerisation of cellulose and hemicellulose by LPMOs. Understanding the mechanism of fungal LPMOs with H_2_O_2_ as a cosubstrate is therefore essential for their use in biorefinery processes. In this investigation, we show that H_2_O_2_ is an efficient cosubstrate for LPMOs. By monitoring both electron transfer from a redox partner protein and product formation, we show that H_2_O_2_ can catalyse multiple (up to 20) LPMO catalytic cycles in the absence of external electron transfer processes. Electron paramagnetic resonance and UV‐Vis spectroscopic methods are used to show that H_2_O_2_ leaves the enzyme in a reduced (Cu^+^) state following catalysis. In this form, the enzyme binds substrate with a higher affinity, has an increased stability and does not require activation by a redox partner protein. We show that substrate binding increases the redox potential of the T2Cu site, an effect that does not stimulate electron delivery from partner proteins but helps to stabilise the reduced state of the enzyme, probably preventing uncoupled reactions from occurring. Furthermore, we identify a number of spectroscopically active species in *Nc*LPMO9C in the absence of the polysaccharide substrate. We hypothesise that these species form part of the hole‐hopping pathway, which may prevent oxidative damage to the enzyme when the substrate is not present.

## Materials and methods

### Materials

Unless otherwise stated, all reagents were of analytical grade and were purchased from Sigma‐Aldrich (Vienna, Austria, and Dorset, UK). Xyloglucan from *Tamarind* seeds (purity 95%) and the xyloglucan‐derived heptasaccharide X_4_Glc_3_ (purity > 90%) were obtained from Megazyme (Bray, Ireland).

### Enzymes

LPMO9C and CDHIIA from *N. crassa* were recombinantly produced in *P. pastoris* X33 as previously described [61] and purified by a two‐step chromatographic procedure employing hydrophobic interaction chromatography (PHE‐Sepharose FF resin) and anion exchange chromatography (DEAE Sepharose FF).

The cytochrome fragment of *N. crassa* CDH IIA was recombinantly produced in *P. pastoris* X‐33 under the control of the methanol‐inducible *AOX1* promoter. Fermentation was done in a 5‐L laboratory fermenter (Eppendorf, Vienna, Austria) by following the *Pichia* fermentation process guidelines from Invitrogen. After an initial glycerol fed‐batch phase to build up biomass, a methanol feed was applied for ca 72 h. The feed was automatically adjusted to maintain an oxygen saturation of 20%. The concentration of *Nc*CYT in the crude extract was 393 mg·L^−1^. The protein was purified by hydrophobic interaction chromatography (PHE‐Sepharose FF resin) and anion exchange chromatography (Q‐Sepharose resin). The purest fraction had an experimentally determined molar absorption coefficient at 420 nm of 89 mm ^−1^ ·cm^−1^. *Nc*CYT was concentrated using centrifugal filters (Centricon; 10 kDa weight cut‐off) and stored at −30 °C.

### EPR Spectroscopy

EPR spectra were recorded using a Bruker ELEXYSYS‐E500/E580 X‐band EPR spectrometer (Bruker GmbH, Rheinstetten, Germany). The microwave power was set to 30 dB, the modulation amplitude set to 5 G, a time constant of 41 ms, a conversion time of 41 ms, a sweep time of 84 s, the receiver gain set to 60 dB and an average microwave frequency of 9.384 GHz. Throughout the measurements, a temperature of 20 K was maintained via an Oxford Instruments ESR900 helium flow cryostat coupled to an ITC503 controller from the same manufacturer. EPR experiments were carried out at 20 K and employed 0.2 mW microwave power, 100 kHz modulation frequency and 5 G (0.5 mT) modulation amplitude. All EPR samples were prepared in an anaerobic glove box (O_2_ < 2 ppm) and placed in 4‐mm Suprasil Quartz EPR Tubes (Wilmad‐LabGlass, NJ, USA). Tubes were sealed with a Suba‐Seal rubber stopper under anaerobic conditions and immediately frozen in liquid N_2_ to prevent reoxidation.

### Redox potentiometry

The redox potential of the T2Cu in *Nc*LPMO9C was determined by electrochemical titration against ascorbate in an anaerobic glove box (O_2_ < 2 ppm). The reaction contained 200 µm LPMO and a redox mediator mix containing 1‐methoxy‐5‐methylphenazinium methyl sulfate (2 µm), methyl viologen dichloride hydrate (0.3 µm), potassium ferricyanide (2 µm), 2‐hydroxy‐1,4‐naphthoquinone (7 µm), N,N,N′,N′‐tetramethylethylenediamine (2 µm) and benzyl viologen (1 µm) to establish communication between the electrode and the active site of the enzyme. The titration was also carried out in the presence of 3.3 mg·mL^−1^ xyloglucan. After each addition of ascorbate, the electrode potential was allowed to stabilise. The electrochemical potential of the solution was measured using a Thermo Orion ORP electrode at 30 °C and corrected by a factor of + 207 mV to convert values to the standard hydrogen electrode (SHE). After each titration step, a sample of 250 μL was withdrawn for EPR analysis. Samples were prepared under anaerobic conditions as described above. Redox titrations were analysed by the Nernst equation using the Origin Pro Software.

### UV‐Vis spectra

UV‐Vis spectra were recorded on an Agilent 8453 diode array spectrophotometer (Santa Clara, CA, USA) placed in an anaerobic glove box (Belle Technology, Weymouth, UK). The measurement cell was maintained at 25 °C during all measurements using an external thermostat. All spectra were measured in 50 mm sodium phosphate buffer, pH 6, using quartz microcuvettes with a total volume of 200 µL and a path length of 1 cm. Buffers and substrate solutions were kept inside the glove box overnight to ensure oxygen‐free conditions.

### Stopped‐flow spectroscopy

Rapid mixing experiments were performed with an SX‐20 stopped‐flow spectrophotometer (Applied Photophysics, Leatherhead, UK) equipped with a diode array detector. The instrument was placed inside an anaerobic glove box (Belle Technology) to avoid reoxidation reactions by molecular oxygen. All reactions were performed in 50 mm sodium phosphate buffer, pH 6. LPMO (100 µm) was stoichiometrically reduced with 50 µm ascorbate and rapidly mixed with a 50‐fold excess (2500 µm) of H_2_O_2_. The temperature of the measurement cell was maintained at 4°C during all measurements. Electron transfer between CDH and LPMO9C in the presence of varying amounts of xyloglucan was probed in sequential mixing mode. Initially, CDH was stoichiometrically reduced with cellobiose and mixed with a fivefold excess of LPMO9C. Varying amounts of XG (0–3 mg·mL^−1^) were added to the LPMO solution. These measurements were carried out at 30°C in an anaerobic glove box. Stopped‐flow traces were fit to exponential functions using the Pro Data software suite (Applied Photophysics). Observed rate constants are presented as an average of three measurements ± 1 SD.

### Determination of reducing ends

Reducing ends generated by *Nc*LPMO9C during oxidoreductive degradation of XG were measured with the colorimetric Somogyi–Nelson assay carried out in the 96‐well plate format. Reagents were prepared as described earlier [62]. In total, 100 µL of the copper–carbonate–tartrate solution (Somogyi solution) was mixed with 100 µL of the sample solution and heated to 100 °C for 15 min in a heating block. After cooling the reaction for 5 min on ice, 100 µL of Nelson solution was added. Precipitates were removed by centrifugation, and 250 µL of this solution was measured at 540 nm in a Perkin Elmer EnSpire Multimode plate reader. A calibration standard was prepared with the XG‐derived heptasaccharide X_4_Glc_3_.

## Conflict of interest

The authors declare no conflict of interest.

## Author contributions

DK and TMH planned the experiments and wrote the manuscript. MS performed EPR experiments and analysed the data. DK, EB, DJH, KK and TMH collected and evaluated stopped‐flow and UV‐Vis data; and DK, TMH, APG, RL and NSS designed and evaluated the experiments.
